# The Guest Hospital, Dudley

**Published:** 1918-09-28

**Authors:** Arthur Bird

**Affiliations:** The Guest Hospital and Eye Infirmary, Dudley


*To the Editor of* THE HOSPITAL.

### The Guest Hospital, Dudley.

*To the Editor of* THE HOSPITAL.

SIR,—If the coward without a name who makes reflections on me and my assistant in the letter you published last week will come into the open I will deal with him, or her.

I have been secretary here for twenty-five years, and my record speaks for itself.

The astonishing thing to me is that a paper of the standing of THE HOSPITAL, to which officials look as their friend, should publish slanderous rubbish.—Yours faithfully, ARTHUR BIRD.

The Guest Hospital and Eye Infirmary, Dudley,
September 11, 1918.

[We publish the above letter in the hope that it may bring the controversy to a head. There has been too much indefiniteness all round, and Mr. Bird's challenge invites a reply, as do the criticisms which he abuses, but has not answered.—ED. THE HOSPITAL.]

